# Association between aortopulmonary collateral flow and clinical status late after Fontan operation

**DOI:** 10.1186/1532-429X-13-S1-P196

**Published:** 2011-02-02

**Authors:** Ashwin Prakash, Rahul Rathod, Andrew J Powell, Yuli Kim, Puja Banka, Tal Geva

**Affiliations:** 1 Children's Hospital Boston, Boston, MA, USA

## Introduction

Excessive aortopulmonary collateral (APC) flow is hypothesized to negatively affect outcome after a Fontan operation. Lack of an accurate and readily applied technique to measure APC flow has hindered research on this issue.

## Purpose

We used a recently described cardiac MRI (CMR) method to quantify APC flow in a large cohort of late Fontan survivors, and evaluate its relationship with clinical outcomes.

## Methods

We performed a retrospective review of patients late after Fontan operation who underwent CMR measurement of APC flow at our institution from 2003 through 2009. APC flow was quantified as either aortic flow minus total caval flow or aortic flow minus sum of branch pulmonary artery flow, and was indexed to body surface area (BSA). Patients with a patent fenestration or baffle leak were excluded.

## Results

APC flow was quantified in 78 patients (age 21.8±10 years, 64% male) 15.9±7 years after Fontan operation. Median APC flow was 0.43 L/min/m^2^ (interquartile range 0.13-0.79 L/min/m^2^), accounting for a median of 15% of systemic flow (interquartile range 4-28%). Excluding patients with >mild valve regurgitation (n=10), higher APC flow/BSA was associated with larger ventricular end-diastolic volume (EDV)/BSA (r=0.4, p=0.001). APC flow/BSA was not associated with age at CMR, age at Fontan, history of systemic-pulmonary artery shunt (n=61) or bidirectional Glenn shunt (n=35), history of catheter-based occlusion of APCs (n=10), congestive heart failure (n=21), atrial or ventricular arrhythmias (n=54), >mild atrioventricular valve regurgitation (n=10), ejection fraction, and peak oxygen consumption (VO2) on maximal exercise stress test (n=43) within 1 year of CMR.

## Conclusions

In this cohort of late survivors of a Fontan operation, APC flow measured by CMR comprised a significant proportion of cardiac output and was associated with larger BSA-adjusted ventricular volume. It did not correlate with surgical history, history of catheter-based occlusion of APCs or clinical outcomes. Continued follow-up is required to further determine the long-term relationship between APC flow and clinical outcomes.

